# Maternal Prepregnancy Obesity Affects Foetal Growth, Birth Outcome, Mode of Delivery, and Miscarriage Rate in Austrian Women

**DOI:** 10.3390/ijerph20054139

**Published:** 2023-02-25

**Authors:** Katharina Syböck, Beda Hartmann, Sylvia Kirchengast

**Affiliations:** 1 Department of Evolutionary Anthropology, University of Vienna, 1030 Wien, Austria; 2 Clinic Donaustadt, 1220 Wien, Austria

**Keywords:** maternal obesity, birthweight, foetal growth, pH value, caesarean section, birth outcome, miscarriage, preterm birth

## Abstract

The increasing obesity rates among women of reproductive age create a major obstetrical problem as obesity during pregnancy is associated with many complications, such as a higher rate of caesarean sections. This medical record-based study investigates the effects of maternal prepregnancy obesity on newborn parameters, birth mode, and miscarriage rate. The data of 15,404 singleton births that had taken place between 2009 and 2019 at the public Danube Hospital in Vienna were enrolled in the study. Newborn parameters are birth weight, birth length, head circumference, APGAR scores, as well as pH values of the arterial and venous umbilical cord blood. In addition, maternal age, height, body weight at the beginning and the end of pregnancy, and prepregnancy body mass index (BMI) (kg/m^2^) have been documented. The gestational week of birth, the mode of delivery, as well as the number of previous pregnancies and births, are included in the analyses. Birth length, birth weight, and head circumference of the newborn increase with increasing maternal BMI. Furthermore, with increasing maternal weight class, there tends to be a decrease in the pH value of the umbilical cord blood. Additionally, obese women have a history of more miscarriages, a higher rate of preterm birth, and a higher rate of emergency caesarean section than their normal-weight counterparts. Consequently, maternal obesity before and during pregnancy has far-reaching consequences for the mother, the child, and thus for the health care system.

## 1. Introduction

The prevalence of overweight and obese people is increasing drastically all over the world [1]. Obesity is no longer only a typical problem in developed countries, it is also growing in developing and emerging nations [2,3]. For that reason, it is already being considered an “obesity pandemic”. With the increasing obesity rates worldwide, a rise in various other diseases associated with obesity is also occurring. Several studies show that the risk of cardiovascular diseases, metabolic diseases such as diabetes type 2, and various cancers increase significantly with obesity [4,5]. In addition, fertility and reproductive success are affected by obesity in both men and women [6,7]. In most countries, women have higher obesity rates than men, with less educated women at two to three times higher risk than those with more education [8]. These high obesity rates are especially serious in women of reproductive age, as overweight and obesity before and during pregnancy are associated with many complications, such as preeclampsia [9], higher caesarean section rates [10,11,12], macrosomia [13], and foetal acidosis [13,14], just to name a few. This is not only problematic for the women themselves, but also for the foetus or newborn, and the consequences can be far-reaching [15,16,17,18,19,20,21,22]. At first, obesity reduces the chance of successful fertilisation, especially in assisted fertilisation, such as in vitro fertilisation. During pregnancy, the risk of miscarriage is higher in obese women [23,24], this is especially true after assisted fertilisation [25]. Even after successful spontaneous conception, pregnancy in obese women is associated with many risks, not only for the mother but also for the newborn [26]. Obesity before and during pregnancy dramatically increases the risk of miscarriage, preterm birth, infant mortality, and stillbirth [27,28], but also of congenital defects, such as spina bifida, cleft lip and palate, hydrocephalus, and heart defects such as septal anomalies [29,30]. On the other hand, maternal obesity, as excessive gestational weight gain, enhances foetal growth and may result in larger head circumferences and macrosomia of the foetus [31,32,33].

A special problem is represented by the birth itself [34]. Some studies showed that the risk of oxygen deficiency during birth and related adverse outcomes was increased in newborns of overweight or obese women [14,35]. Oxygen deficiency during birth and perinatal asphyxia are associated with cerebral disorders, and thus increase the risk for impaired neurological development [36,37,38,39] and, in the worst case, for neonatal death [39]. There are also long-term consequences of oxygen deprivation such as cerebral palsy, a spastic dysfunction of locomotion [40]. Despite oxygen deficiency, maternal obesity is often associated with low APGAR scores, indicating severe neonatal stress and a poor adaptation to the postnatal environment of the newborn [41]. Furthermore, maternal obesity is associated with an increased caesarean section rate [11,12,15,34]. Caesarean sections in Class III obese women, however, are especially risky and technically difficult to perform. Accordingly, pregnancies in obese women are considered high-risk pregnancies, that pose special risks for mothers and foetuses.

The present study focuses on the association patterns between maternal weight status and pregnancy outcome in Vienna, Austria over the last 14. years. Austria is not only one of the richest countries of the European Union, the medical health care system is highly developed. All Austrian residents have social insurance that covers all medical costs in public hospitals. During the 1970s, the sophisticated system of the so-called “mother-child passport” was introduced, which guarantees at least three prenatal examinations starting at the 8th gestational week, and eight postnatal check-ups of the child by paediatricians between birth and the age of 4 years free of charge. The completion of all 11 medical examinations is rewarded with a financial premium by the government. The introduction of the mother-child passport helped to make pregnancies and birth much safer. Consequently, pregnant women and newborns are well cared for by the public health service. On the other hand, the prevalence of overweight and obese among young women of reproductive age is steadily increasing in Austria. In 2014, about 13% of 15 to 29 years old females were overweight and about 6% were obese; only 5 years later, however, the prevalence of overweight and obesity increased to 16.1% and 6.7%, respectively, in this age range. A similar trend is found for women aged between 30 and 44 years. In 2014, 21% of women in this age group were overweight and about 8% were obese. In 2019, the numbers increased to 25% and 12%, respectively [42]. Despite the increasing prevalence of overweight and obesity in Austria, the overweight and obesity rates, among women of reproductive age are still lower than in other countries such as the United States [43]. Overweight and obesity during reproductive age is a matter of concern in Austria because rising obesity rates among pregnant women not only place a short-term burden on the health care system, but can have long-term impacts, as the consequences of obesity during pregnancy can have long-lasting effects on both mother and child. In the present study, we examine association patterns between maternal weight status and parameters of pregnancy, delivery mode, foetal growth and birth outcome in Vienna, Austria. In detail we tested the following hypotheses:

(1) Overweight or obese mothers are more likely to experience preterm birth (<37 gestational weeks), they have a history of more miscarriages, and a higher rate of caesarean section than normal-weight mothers.
(2) Among term birth (≥37 gestational weeks), the newborns of overweight or obese mothers are larger and heavier, but show lower APGAR scores than newborns of normal-weight mothers.
(3) Among term birth (≥37 gestational weeks), spontaneous delivered newborns of primiparous overweight or obese mothers have a higher risk of oxygen deficiency.

## 2. Materials and Methods

### 2.1. Dataset and Study Design

In this retrospective medical-record-based single-centre study, the data of 15,404 singleton births were included. Newborns with congenital anomalies were excluded. All births had taken place at the public Danube Hospital (Clinic Donaustadt) in Vienna, Austria between 2009 and 2019. This hospital is one of the largest public birth clinics in Vienna. In the first step, we included all singleton births (n = 15,404), independently of the duration of pregnancy and tested the associations between maternal prepregnancy weight status and the history of miscarriages, preterm birth, as well as the mode of delivery. In the second step, we included term birth exclusively (n = 14,444) and analysed the association between maternal prepregnancy weight status and pregnancy outcome, i.e., newborn size and APGAR scores among term birth (≥37 gestational weeks), only. In the third step, only spontaneously delivered term birth of primiparous mothers (n = 5260) were included and the associations between maternal prepregnancy weight status and cord blood pH values were analysed.

The study was conducted according to the guidelines of the Declaration of Helsinki and approved by the Ethics Committee of Vienna (responsible for Public Hospitals) (Protocol number: EK 19-274-VK 18 March 2020).

### 2.2. Newborn Parameters

Newborn parameters include birth weight (in grams) using a digital infant scale, birth length (in cm) using a standard measurement board for infants, and head circumference (in cm) using a standard tape. All measurements were taken immediately after birth by a trained midwife. In this study, macrosomia is defined as a birth weight greater than 4000 g [44]. APGAR scores have been recorded 1, 5, and 10 minutes after birth [45]. In addition, the foetal presentation at birth (cephalic, breech, transverse) is documented. The pH value of the arterial and venous umbilical cord blood, which represent an accurate, reproducible, and objective evaluation of the oxygen deficiency during birth, was measured to one decimal place. Using the pH value of the umbilical cord blood is therefore recommended to evaluate the newborn outcome [46,47]. For correct and adequate testing of the hypothesis that cord blood pH value is associated with maternal weight status, we defined some additional exclusion criteria. First, only pH values >6.4 were considered, since lower values are more likely to be erroneous. Secondly, caesarean sections and preterm births were excluded, since caesarean sections or preterm births could be the actual reasons for oxygen deficiency and thus for a low pH value of the umbilical cord blood. In addition, only primiparous women were included in order to obtain a sample that was as homogeneous as possible, as foetal acidosis depends on parity [14]. Therefore, only births that meet the following criteria are included in the cord blood analysis: primiparous women who experienced vaginal births after the 37th week of pregnancy (i.e., no premature births).

### 2.3. Maternal Parameters

The maternal parameters include maternal age (in years), height (in cm), and body weight at the beginning and at the end of pregnancy (in kg). Trained personnel measured height to the nearest 0.1 cm using a standard anthropometer. Prepregnancy weight was recorded by an interview using the retrospective method, this means that pregnant women were asked about their body weight before pregnancy at the first prenatal examination. In addition, body weight was measured to the nearest 0.1 kg on a balance beam scale, at the first prenatal visit around the 8th week of gestation. As pointed out above, maternal weight was measured again before delivery (at the end of pregnancy). The weight gain during pregnancy was calculated by subtracting prepregnancy weight from body weight before delivery. In addition, the prepregnancy BMI (kg/m^2^) is calculated and classified into the following categories according to the WHO criteria [48]: underweight < 18.50; normal weight 18.50–24.99 (kg/m^2^); overweight 25.00–29.99 (kg/m^2^); Class I and Class II obesity 30.00–39.99(kg/m^2^); Class III obesity > 40 (kg/m^2^). For a more detailed description of the anthropometric methods applied see Kirchengast and Hartmann [49].

### 2.4. Obstetrical Parameters

The following obstetrical characteristics were recorded: preterm birth (<37 gestational weeks), versus term birth (≥37 gestational weeks), mode of delivery (spontaneous vaginal delivery, vacuum extraction/forceps, planned caesarean section, unplanned or emergency caesarean section), foetal presentation at birth (cephalic presentation, breech presentation, transverse presentation), and number of miscarriages. At the first prenatal visit, all mothers were asked concerning previous miscarriages. In addition, the number of miscarriages was calculated as the difference of reported pregnancies and births.

### 2.5. Statistical Analysis

The statistical analysis was carried out with IBM SPSS version 27. The significance level was set to 0.05. After computing descriptive statistics, Kruskal–Wallis *H* tests were performed to test for differences in neonatal and maternal parameters between maternal weight classes. Bonferroni post hoc tests were then conducted to allow pairwise comparisons between all groups. To evaluate the risk of macrosomia, the odds ratios were calculated for each maternal weight class compared to the normal-weight women with the respective 95% confidence interval. To test for an association between maternal weight class and miscarriages, as well as the mode of delivery, Pearson Chi^2^ tests were performed. In addition, the odds ratios for miscarriage were calculated for each maternal weight class compared to the normal-weight women with the respective 95% confidence interval. The relative risk of an emergency caesarean section was calculated for each weight category compared to normal-weight women. A linear regression analyses was computed to test the association patterns between the pH value of the arterial cord blood and maternal body mass index. Binary logistic regression was used to calculate the effect of maternal weight class on the mode of delivery, independent of weight gain during pregnancy, maternal age, and body height.

## 3. Results

### 3.1. Sample Characteristics

Table 1 presents maternal, newborn, and obstetrical characteristics. The average age of the mother was 30.0 years ranging from 15 to 45.5 years. Their average height was 165.4 cm. The average maternal weight before pregnancy was 66 kg, and it was 80 kg at the end of pregnancy. On average, the women in this sample gained 22.4% of their prepregnancy body weight during pregnancy. A total of 952 women (6.2%) corresponded to the definition of underweight, showing a BMI less than 18.50 kg/m^2^, 61.0% were normal weight and corresponded to the recommended range of 18.50 to 24.99 kg/m^2^. A further 20.5% were classified as overweight with a BMI between 25 and 29.99 kg/m^2^, 10.7% were obese with a BMI between 30 to 39.99 kg/m^2^, and 1.2% were Class III obese showing a BMI greater than 40 kg/m^2^. The average birth weight in this sample was 3385.6 g, the average birth length was 50.6 cm, and the average head circumference was 34.2 cm. A resulting 11.1% of the newborns were classified as low-weight (<2500 g), and 11% corresponded to the definition of macrosomia (≥4000 g).

As demonstrated in Table 1, the majority of births occurred spontaneously, the most common child presentation was cephalic. More than 45% of the mothers were primiparous. The preterm birth rate was 6.2%. Furthermore, 31.3% of the mothers had experienced at least one miscarriage, 9.9% of the mothers two or more miscarriages. The mean number of miscarriages was 0.5, but one woman experienced 14 miscarriages.

### 3.2. Maternal and Obstetrical Characteristics According to Maternal Weight Status

The maternal age differs significantly between the weight status groups (*p* < 0.001) (Table 2). Subsequent post hoc tests show that only the underweight women differ significantly in age from the other groups, with the underweight mothers being on average two years younger than those in the other weight status groups. Significant differences between the groups can also be recognised with regard to height (*p* < 0.001). However, only the overweight and underweight women differ significantly in terms of height, with the underweight women being on average taller than the overweight ones. Gestational weight gain decreased significantly with increasing weight status (*p* < 0.001.) With the exception of underweight and normal-weight groups, all weight status groups differed significantly from every other group (Table 2).

Preterm birth rate differed significantly (λ = 22.840, d.f. = 4, *p* < 0.001) between maternal weight status groups. The highest rate of preterm birth occurred among obese women, the lowest among normal-weight women. Furthermore, a significant relationship (λ = 35.120, df = 4, *p* < 0.001) occurred between the history of miscarriages and maternal weight status. Underweight and normal-weight women do not differ significantly in miscarriage risk. Between overweight women and normal-weight women, however, significant differences in miscarriage risk occur. The risk of miscarriage is increased by about 1.2 times in overweight (95% CI 1.09–1.29) and obese women (95% CI 1.06–1.33) compared to normal-weight women. The effect is even stronger in Class III obese women, with a 1.8 times higher risk of miscarriage (95% CI 1.33–2.37) compared to normal-weight women (Table 2).

A significant relationship can be found between maternal weight classes and mode of delivery (λ = 81.777, d.f. = 12, *p* < 0.001). It can be seen that obese and Class III obese women in particular have more caesarean deliveries (planned and emergency) and fewer vaginal, spontaneous deliveries (Table 3). The risk of emergency caesarean section increases continuously from 1.2 times higher risk (95% CI 1.07–1.41) in overweight women to 2.2 times higher risk (95% CI 1.44–3.31) in Class III obese women compared to normal-weight women, while there is no significant difference between underweight and normal-weight women in terms of the risk of emergency caesarean deliveries.

A binary logistic regression model is calculated to show the independent effects of maternal BMI on the mode of delivery. It shows that maternal BMI has an independent significant effect (*p* < 0.001) on the occurrence of emergency caesarean sections. In addition to the BMI, weight gain during pregnancy, maternal age, and neonatal head circumference have a positive, significant, and independent effect on emergency caesarean section rates. Maternal height, gestational week, and birth weight are significantly negatively associated with the caesarean section rate (Table 3).

### 3.3. Newborn Characteristics According to Maternal Weight Status

With increasing maternal weight status, the newborns become significantly longer (*p* < 0.001). Post hoc tests show that only the birth lengths of the newborns of normal-weight and overweight women do not differ significantly, while there are significant differences between all other weight status groups. Birth weight also shows significant differences between the weight status groups (*p* < 0.001) with a tendency for birth weight to increase as the mother’s BMI increases. The highest birth weight was found among obese mothers. Pairwise comparisons show that the birth weights of the children of underweight and normal-weight women are each significantly different from every other group. Within the overweight, obese, and Class III obese women, however, no significant birth weight differences can be detected. Head circumference also differs significantly between the weight status groups (*p* < 0.001), in which the head circumference tends to increase as the mother’s BMI increases. More precisely, the head circumference of children of underweight women is significantly smaller than in all other groups and the head circumference of children of normal-weight women is significantly smaller than that of children of overweight and obese women (Table 4).

The risk of newborn macrosomia (>4000 g) increases significantly with increasing maternal weight status. While the newborns of underweight women have a significantly (*p* < 0.001) lower risk of macrosomia (OR = 0.45; CI 95% 0.31–0.61) than those of normal-weight women, neonates of overweight women have a 1.37-fold (CI 95% 1.21–1.55) higher risk of macrosomia compared to those of normal-weight women (*p* < 0.001). The effect is even more pronounced in the newborns of obese (*p* < 0.001) and Class III obese women (*p* < 0.001). Here, the risk of macrosomia increases by a factor 1.88 (CI 95% 1.62–2.17) and 2.0 (CI 95% 1.38–2.93), respectively, compared to newborns of normal-weight women.

Furthermore, we found a significant association between APGAR scores after 1, 5, and 10 min and maternal weight status. The APGAR scores decreased significantly with increasing maternal weight status.

The pH value of the arterial cord blood differed significantly across maternal weight status categories (*p* < 0.001, H = 26.601). Subsequent Bonferroni corrections show that obese women differ significantly from underweight and normal-weight women (*p* = 0.005 and *p* = 0.011, respectively). In addition, overweight women also differ significantly from underweight and normal-weight women (*p* = 0.012 and *p* = 0.014, respectively). Furthermore, according to the results of a linear regression analysis, the pH value of the arterial cord blood decreases with increasing maternal BMI (B = 7.258, β = −0.066, *p* < 0.001, R*^2^* = 0.004). Although highly significant, the coefficient of determination is relatively low. Only 0.4% of the variance of the pH value can be explained by the maternal BMI. The differences in venal blood pH values between the weight status groups are not significant; nevertheless, there is still a trend apparent that the pH value of the venous cord blood decreases with increasing maternal weight class (Table 4).

## 4. Discussion

The worldwide trend of rising obesity rates is particularly serious among women of reproductive age, as obesity before and during pregnancy is associated with various severe—sometimes long-term—complications for mother and child [19,22,26,50,51,52,53]. This study in particular focuses on newborn parameters, cord blood pH values, miscarriage rate, mode of delivery, and the respective effect of maternal obesity on each of them in an Austrian sample. Altogether the data of 15,404 singleton births taking place at the public Danube hospital in Vienna, Austria, were included in the analysis. A total of 20.5% of the mothers corresponded to the definitions of overweight (BMI 25.00–29.99 kg/m^2^), and 10.7% to the definition of obesity(BMI 30.00–39.99 kg/m^2^). Furthermore, 1.2% of the included women showed a prepregnancy BMI above 40.00 kg/m^2^. These overweight and obesity rates are typical of Austria [42], although only an urban sample was analysed.

The first hypothesis that overweight or obese mothers are more likely to experience preterm birth (<37 gestational weeks), to have a history of more miscarriages, and a higher rate of caesarean section than normal-weight mothers could be verified. A significant association between weight status and history of miscarriage was detected (*p* < 0.001), with overweight and obese women having experienced more miscarriages than normal-weight women. The risk of ever having suffered a miscarriage is 1.2 times higher in overweight women and 1.8 times higher in Class III obese women than in normal-weight women. This finding is in accordance with previous studies which have shown that with higher maternal BMI, the risk of miscarriage increases [24]. A direct causality between obesity and increased miscarriage rate, however, cannot be concluded from our results due to the lack of data on weight status at the time of miscarriage. Furthermore, the prevalence of preterm birth (≤37 gestational weeks) was significantly highest (8.5%) among mothers who were overweight before pregnancy, while the lowest rate (5.7%) occurred among normal-weight ones. Concerning breech presentation, however, we found no significant association with maternal weight status.

Considering the association between mode of delivery and maternal prepregnancy obesity, our results are largely consistent with the previous literature [11,15]. The hypothesis that birth mode is related to maternal weight status was thus verified. A significant relationship between maternal weight class and mode of delivery could be shown (*p* < 0.001). It is particularly important to clarify to what extent maternal weight is related to emergency caesarean section rates. Emergency caesarean deliveries are those C-sections that are not planned and that require an acute change from a vaginal delivery to a caesarean section. Since emergency caesarean sections are acutely medically necessary interventions, those types of C-sections are in the focus of this study. As maternal BMI increases, the emergency caesarean section rate also increases. It is particularly noticeable that the risk for an emergency caesarean section very strongly relates to the severity of obesity. While overweight women have a 1.2-fold higher risk and obese women a 1.4-fold higher risk, Class III obese women have more than twice the risk (2.1-fold higher) of experiencing an emergency caesarean section compared to normal-weight women. This is a very abrupt increase in risk between obese and Class III obese women. These results are roughly in line with the findings of Chu et al. [27], although the calculated C-section risks of their study are even slightly higher.

The second hypothesis, that newborns of overweight or obese mothers are larger and heavier but show lower APGAR scores than newborns of normal-weight mothers, could be verified. Considering birth length, birth weight, and head circumference, it is evident that as maternal prepregnancy BMI weight class increases, the neonate has significantly larger dimensions. This is largely consistent with the previous literature [16,34,35]. Nevertheless, previous studies have shown that maternal obesity, and especially Class III obesity, can not only cause relatively large newborns and macrosomia, but can also be responsible for low birth weight and even foetal growth retardation. Maternal obesity may thus have multidirectional and opposing effects on birth weight [13]. In our sample, the risk of macrosomia increases with increasing weight status. While the risk for macrosomia is 1.4 times higher in overweight women than in normal-weight women, the risk in obese women is almost twice as high compared to normal-weight women.

The precise mechanisms of how maternal obesity affects foetal growth are not yet fully understood. Certainly, maternal obesity exposes the foetus to a different hormonal and external environment. The complex interaction of environment and genes can lead to enhanced growth in the foetus resulting in macrosomia [54], as found in the present study. Macrosomia, in turn, is associated with several complications, including increased risk of caesarean section, extended stay in hospital, chorioamnionitis [55], and many others. The results of the present study suggest, that maternal overweight and especially obesity before and during pregnancy, affect foetal growth and consequently newborn size.

The third hypothesis that maternal prepregnancy weight status is related to cord blood pH values, however, could only be partially verified. According to previous studies, overweight and obesity are associated with foetal acidosis [14]. A valid indicator of such oxygen deficiency during birth is the pH value of the umbilical cord blood [14]. In our study, the association of cord blood pH value and maternal weight status is only partially consistent with the previous literature. Although the regression line between (arterial) pH values and maternal BMI shows a negative slope, indicating a negative correlation between the two variables, the corresponding R^2^ is relatively small (0.004), maternal BMI can explain only a very small amount of variance in pH values. Dividing BMI again into the respective weight categories, there is a tendency for median values of pH to decrease with increasing maternal weight categories, with normal-weight women having a mean of 7.24 and Class III obese women having a mean of 7.21. Both obese and overweight women differ significantly from normal-weight and underweight women in arterial cord blood pH values. The question that arises is why, contrary to previous literature such as [14], the other groups do not differ significantly from each other with respect to this characteristic in this sample. This is likely due to the small sample sizes of some weight classes. The group of Class III obese women (n = 47), especially, is strongly underrepresented in our study. Looking now at the pH values of the venous cord blood, there are no significant differences at all in the pairwise comparisons between the groups. Although the same trend as in the analysis of the pH value of the arterial blood is observed, namely a decrease of the pH value with increasing maternal weight class, these results are not significant.

To sum it up, the results of the present study suggest, that maternal overweight, and in particular maternal obesity even before pregnancy, has a negative effect on birth outcome and on delivery, resulting in an increased risk of emergency caesarean sections. Considering the rising obesity rates among women of reproductive age worldwide, these findings are of particular concern. Increasing obesity rates are mostly the result of profound changes in lifestyle patterns, characterised by a reduction of physical activity and an increase of high calorie intake. Increasing obesity rates, and also the trend to postpone motherhood, have led to an increase in emergency caesarean section rates [12]. In our study, a binary logistic regression model shows an independent effect of BMI on the mode of delivery (*p* < 0.001), respectively, on the incidence of emergency caesarean section. Maternal obesity is clearly associated with an increased risk of emergency caesarean section, and poses in this way a special burden for health care systems, as they are associated with high costs and effort [56]. Surgeries including C-sections for severely obese individuals are a much greater expense for health care professionals and on average, obese individuals also require longer hospital stays [57,58,59]. In Class III obese women especially, a caesarean section presents great technical difficulties, some of which can have severe life-threatening consequences [60,61]. The problems range from difficulty in anaesthesia and finding the epidural space, to difficult conditions in transporting patients and increased numbers of staff required [11]. This causes another burden on the health care system. In order to achieve a sustainable reduction of the emergency caesarean section rate, i.e., the rate of those caesarean sections that are medically necessary, a reduction in the obesity rate among women of childbearing age would be of major importance.

Although the results of our study correspond to the finding of previous investigations, the limitations of our study should not go unmentioned. A major limitation is the lack of information regarding socioeconomic status and educational level of the mothers. It is well documented that maternal educational level and socioeconomic status are strongly related to reproductive performance and birth outcome, but also the prevalence of overweight and obesity [62,63,64]. Nevertheless, many previous studies show that the negative effects of maternal obesity during pregnancy on the newborn are independent of socioeconomic status [19]. Therefore, it still makes sense to examine the relationships between maternal obesity and neonatal parameters, even without information on social status. We are aware of this limitation; however, as pointed out in the methods section, this is a medical record-based study without access to socioeconomic data.

Another important limitation is the use of BMI as a measure of weight status. There is no doubt, that the BMI is a widely used and accepted indicator to evaluate weight status in adults. Nevertheless, the BMI has some limitations that cannot be ignored. Since BMI is only a weight to length measure and does not take body composition into account, no distinction is made between lean mass and fat mass. A person with a high muscle mass therefore also has a high BMI and might thus incorrectly be classified as overweight or even obese. Vice versa, the BMI of a person with high fat mass but low lean mass is also not sufficiently informative to a certain extent [65]. Nevertheless, the BMI is a practical measure to determine the weight status because, unlike DEXA (=dual-energy X-ray absorptiometry), air displacement plethysmography, and many other sophisticated methods, its use is cheap, easy to survey, non-invasive, and also safe.

Another limitation represents the documentation of previous miscarriages. The difference between the number of pregnancies and the number of actual births alone, of course, as an indicator of the miscarriage rate says nothing about intentional and wanted abortions. Since no information on wanted abortion rates is available, they cannot be considered in this analysis. Therefore, only the difference between the number of pregnancies and births is used as an indicator for miscarriage. Although induced abortions are legal in Austria, there is no official documentation of the total number of abortions.

The strength of this study is the huge sample size, i.e., more than 15,000 singleton births were included in the analysis.

## 5. Conclusions

The present study clearly demonstrates the various associations between maternal overweight or obesity and birth outcome as well as obstetrical parameters. Obesity during pregnancy is associated with many adverse effects, such as an increased miscarriage rate, a higher rate of emergency caesarean sections, lower pH values of the umbilical cord blood, and also greater newborn size and a higher risk of macrosomia. Besides acute birth complications, far-reaching long-term consequences for mother and child may arise from maternal obesity. It is therefore of extreme importance to address the issue at all levels of the health care system, but also at all levels of society. These activities should include prevention strategies as well as weight loss therapies that help obese women to sustainably achieve a healthy body weight. To do this, the problem must be considered in its entirety and not at the level of the individuals themselves. Here, it is up to decision-makers to act. Effective measures should be taken by politicians, such as stricter advertising rules, especially for products consumed by children, price regulation such as a sugar tax, and more awareness and education in schools. Bold actions are needed to get the complex phenomenon of obesity under control.

## Figures and Tables

**Table 1:** Sample characteristics using descriptive statistics.

Maternal and Newborn Parameters	Mean (SD)	Range	*n*
Age (years)	30.0 (5.6)	15.0–45.5	15,405
Body height (cm)	165.4 (6.3)	140–193	15,405
Prepregnancy weight (kg)	66.0 (14.4)	35–162	15,400
End of pregnancy weight (kg)	80 (14.6)	43–172	14,406
Gestational weight gain (kg)	14.0 (6.0)	−30–43	14,403
Prepregnancy BMI (kg/m^2^)	24.08 (4.98)	13.39–61.33	15,353
<18.50 kg/m^2^			952 (6.2%)
18.50–24.99 kg/m^2^			9398 (61.0%)
25.00–29.99 kg/m^2^			3160 (20.5%)
30.00–39.99 kg/m^2^			1653 (10.7%)
≥40 kg/m^2^			190 (1.2%)
Number of pregnancies	2.3 (1.4)	1–15	15,405
Number of births	1.8 (0.9)	1–12	15,405
Number of miscarriages	0.5 (0.8)	0–14	15,405
First-time mothers			7098 (46.1%)
Preterm birth			953 (6.2%)
Birth mode			
Spontaneous vaginal delivery			12,128 (78.8%)
Vacuum extraction/Forceps			820 (5.3%)
Planned Caesarean section			1099 (7.1%)
Emergency Caesarean section			1350 (8.8%)
Birth presentation			
Cephalic presentation			14,523 (94.7%)
Breech presentation			757 (4.9%)
Transverse presentation			47 (0.4%)
Newborn sex			
female			7449 (48.4%)
male			7974 (51.6%)
Birth length (cm)	50.6 (2.6)	28.0–56.0	15,382
Head circumference (cm)	34.2 (1.7)	21.5–53.0	15,375
Birth weight (g)	3385.6 (540.0)	470–5350	15,405
<2500 g			740 (11.1%)
2500–3999 g			12,970 (84.2%)
≥4000 g			1695 (11.0%)
APGAR value 1 min			
APGAR value 5 min			
APGAR value 10 min			
pH value (arterial cord blood)	7.3 (0.1)	6.5–7.7	5554
pH value (venous cord blood)	7.3 (0.2)	6.9–7.7	5470

**Table 2:** Maternal parameters according to weight classes and obstetrical characteristics according to weight classes. Significance levels.

	Maternal Weight Status					
	Underweight	Normal Weight	Overweight	Obese	Class III Obese	*p*-Value
	Mean (SD)	Mean (SD)	Mean (SD)	Mean (SD)	Mean (SD)	
Age (years)	28.1 (5.9)	30.0 (5.5)	30.3 (5.6)	30.3 (5.5)	30.5 (5.4)	<0.001 ^a,b,c,d^
Height (cm)	165.7 (6.2)	165.6 (6.3)	165.1 (6.4)	165.1 (6.2)	166.3 (5.8)	<0.001 ^b^
PPW (kg)	48.5 (4.4)	59.6 (6.4)	74.1(6.9)	90.9 (10.2)	119.8 (12.4)	/
Weight gain (kg)	14.8 (5.6)	14.8 (5.3)	13.5 (6.2)	10.8 (7.2)	7.3 (7.2)	<0.001 ^b,c,d,e,f,g,h,i,j^
	%	%	%	%	%	
Preterm birth	7.6%	5.7%	6.0%	8.5%	6.3%	<0.001
miscarriage	29.5%	30.0%	33.8%	33.7%	43.2%	<0.001
Breech presentation	5.3%	5.2%	4.4%	4.6%	4.8%	0.137
Planned section	6.0%	6.5%	7.5%	10.0%	13.7%	<0.001
Emergency section	6.9%	8.1%	9.7%	11.0%	14.7%	<0.001

Kruskal–Wallis *H* test (and Bonferroni corrections) and Chi-squares. Legend: PPW = prepregnancy weight. Dunn–Bonferroni post hoc test: significant difference between: ^a^ = weight status 1 and 2; ^b^ = weight status 1 and 3; ^c^ =weight status 1 and 4; ^d^ = weight status 1 and 5; ^e^ = weight status 2 and 3; ^f^ =weight status 2 and 4; ^g^ = weight status 2 and 5; ^h^ = weight status 3 and 4; ^i^ = weight status 3 and 5; ^j^ = weight status 4 and 5.

**Table 3:** Maternal and foetal parameters associated with emergency caesarean section. Binary logistic regression.

Independent Variables	B (SE)	*p* Value	Exp(B)	95% Confidence Interval		R^2^
				Lower Value	Upper Value	
Maternal BMI	0.051 (0.006)	<0.001	1.053	1.041	1.065	0.057
Weight gain	0.048 (0.005)	<0.001	1.049	1.039	1.060	
Maternal age	0.038 (0.005)	<0.001	1.039	1.028	1.050	
Maternal height	−0.048 (0.005)	<0.001	0.953	0.944	0.962	
Gestational week	−0.047 (0.021)	0.022	0.954	0.916	0.993	
Birth weight	−0.001 (<0.001)	<0.001	0.999	0.999	0.999	
Head circumference	0.151 (0.023)	<0.001	1.163	1.112	1.21	

**Table 4:** Newborn parameters according to maternal weight classes Kruskal–Wallis *H* test (and Bonferroni corrections) and comparison of significance levels.

	Maternal Weight Status					
	Underweight	Normal Weight	Overweight	Obese	Class III Obese	*p*-Value
	Mean (SD)	Mean (SD)	Mean (SD)	Mean (SD)	Mean (SD)	
BL (cm)	50.3 (1.9)	50.9 (2.1)	51.2 (2.2)	51.3 (2.1)	51.2 (2.0)	<0.001 ^a,b,c,d,e,f,g^
BW (g)	3246.9 (430.0)	3430.3 (430.1)	3516.9 (465.5)	3569.1 (496.4)	3547.6 (522.2)	<0.001 ^a,b,c,d,e,f,g^
HC. (cm)	33.9 (1.3)	34.3 (1.4)	34.5 (1.5)	34.6 (1.5)	34.7 (1.7)	<0.001 ^a,b,c,d,e,f,g^
APGAR 1 min	9.16 (0.99)	9.10 (0.98)	9.05 (0.98)	8.95 (1.12)	8.72 (1.28)	<0.001 ^b,c,d,e,f,g,i,j^
APGAR 5 min	9.85 (0.56)	9.82 (0.62)	9.79 (0.67)	9.72 (0.82)	9.65 (0.77)	<0.001 ^c,d,f,g^
APGAR 10 min	9.96 (0.44)	9.95 (0.32)	9.94 (0.41)	9.92 (0.36)	9.90 (0.39)	<0.001 ^d,g^
pH value arterial blood	7.24 (0.08)	7.23 (0.08)	7.22 (0.08)	7.22 (0.09)	7.21 (0.07)	<0.001 ^d,g,i,j^
pH value venous blood	7.30 (0.07)	7.30 (0.08)	7.29 (0.08)	7.29 (0.08)	7.29 (0.07)	0.765

Legend: Bl = birth length, BW = birthweight, HC = head circumference, Dunn–Bonferroni post hoc test significant difference between: ^a^ = weight status 1 and 2; ^b^ = weight status 1 and 3; ^c^ =weight status 1 and 4; ^d^ = weight status 1 and 5; ^e^ = weight status 2 and 3; ^f^ =weight status 2 and 4; ^g^ = weight status 2 and 5; ^i^ = weight status 3 and 5; ^j^ = weight status 4 and 5.

## Data Availability

Restrictions apply to the availability of the data used in this study-Data were obtained from medical records of the Danube Hospital, Vienna and are exclusively with the permission of the Wiener Gesundheitsverbund.
